# Diagnostic strategies for posttraumatic osteomyelitis: a survey amongst Dutch medical specialists demonstrates the need for a consensus protocol

**DOI:** 10.1007/s00068-017-0783-9

**Published:** 2017-03-22

**Authors:** G. A. M. Govaert, A. W. J. M. Glaudemans, J. J. W. Ploegmakers, A. R. Viddeleer, K. W. Wendt, I. H. F. Reininga

**Affiliations:** 1 Department of Surgery, Subdivision of Trauma Surgery, University of Groningen, University Medical Center Groningen, P.O. Box 30001, 9700 RB Groningen, The Netherlands; 2 Department of Nuclear Medicine and Molecular Imaging, University of Groningen, University Medical Center Groningen, P.O. Box 30001, 9700 RB Groningen, The Netherlands; 3 Department of Orthopaedics, University of Groningen, University Medical Center Groningen, P.O. Box 30001, 9700 RB Groningen, The Netherlands; 4 Department of Radiology, University of Groningen, University Medical Center Groningen, P.O. Box 30001, 9700 RB Groningen, The Netherlands; 5 Present Address: Department of Trauma Surgery, University Medical Center Utrecht, Internal mail no G04.228, P.O. Box 85500, 3508 GA Utrecht, The Netherlands

**Keywords:** Osteomyelitis, Trauma, Infection, Fracture, Medical imaging, Radiology, Nuclear imaging, Protocol

## Abstract

**Introduction:**

Posttraumatic osteomyelitis (PTO) is a feared complication after surgical fracture care. Late diagnosis can result in interrupted and prolonged rehabilitation programmes, inability to work, medical dependency, unnecessary hospital admissions, and high medical and non-medical costs. Primary aim of this study was to assess preferred diagnostic imaging strategies for diagnosing PTO amongst orthopaedic and trauma surgeons, radiologists, and nuclear medicine physicians. Secondary aims were to determine the preferred serum inflammatory marker for diagnosing PTO and the existence of a local hospital protocol to diagnose and manage PTO.

**Materials and methods:**

This study utilised an online survey based on four clinical scenarios, varying from early to late onset of PTO. It was designed to assess individual practitioners’ current preferred diagnostic strategy for diagnosing PTO. Eligible study participants were medical specialists and registrars in orthopaedic and trauma surgery, musculoskeletal (MSK) radiology, and nuclear medicine.

**Results:**

There were 346 responders: 155 trauma surgeons, 102 orthopaedic surgeons, 57 nuclear medicine physicians, and 33 MSK radiologists. Trauma surgeons favour FDG-PET to image PTO, while orthopaedic surgeons prefer WBC scintigraphy. A similar difference was seen between radiologists and nuclear medicine physicians (MRI versus nuclear medicine imaging). CRP was regarded as the most useful serum inflammatory marker. Only one-third of all responders was aware of a local hospital protocol for the treatment of osteomyelitis.

**Conclusions:**

The availability of and awareness towards local protocols to diagnose and treat PTO is poor. The results of this study support the need for future randomised controlled trials on optimal diagnostic strategies for PTO.

## Introduction

The reported incidence of 1–19% of deep infections after surgical fracture care is much higher than in procedures such as elective orthopaedic joint replacement (reported infection rate 0.8–1.2%) [1–4]. This is not surprising, not only because of the typically acute setting in which trauma surgery takes place but also because of numerous other contributing causes. The nature of a fracture (anatomic location, open versus closed, high-energy versus low-energy impact), level of wound contamination in open fractures, systemic inflammatory response due to soft-tissue injury, possible accompanying vascular injury, timing, and duration of surgery, and severity of concomitant injuries (which can require a hasty damage control procedure) are all factors that influence the risk of developing a deep fracture-related infection [1, 3, 5–11]. Diagnosing fracture-related osteomyelitis, also referred to as posttraumatic osteomyelitis (PTO), is challenging and requires in-depth knowledge of the problem as well as a high index of suspicion by the treating medical team [12, 13]. A surgical site infection (SSI) is usually easily recognisable by the four classical signs of infection: calor, dolor, rubor, and tumor. This is rarely the case for a long-standing PTO which can present with a closed wound and no apparent acute signs of infection. Symptoms such as pain and disability to use the affected limb can mimic other differential diagnoses like non-infected non-union, posttraumatic arthrosis or simply symptomatic hardware.

Most recommendations for the best diagnostic workup of PTO are at best level-4 evidence, based on expert opinions and local consensus meetings [7, 14–17]. Serum inflammatory markers, such as leukocyte count (LC), C-reactive protein (CRP) and erythrocyte sedimentation rate (ESR) are widely used, but their diagnostic value for PTO is poorly studied. The same can be concluded for medical imaging modalities. With recent developments in hybrid camera systems, such as Single Photon Emission Computed Tomography combined with Computed Tomography (SPECT-CT) and Positron Emission Tomography combined with CT (PET-CT), there are now more advanced methods to image PTO [17]. These newer techniques achieve a higher diagnostic accuracy by combining pathophysiology with anatomy in a single imaging modality. Although they are already used on a large-scale worldwide, these modern imaging modalities are not yet prospectively studied in large PTO patient populations and, therefore, have not yet been implemented in evidence-based guidelines. Most clinicians acknowledge the fact that every imaging technique has its advantages and disadvantages, and rely on local customised preferences and logistic availability. X-rays and CT are useful to assess the position of metal implants, fracture stability, and bone healing. Osteomyelitis can sometimes be detected by periosteal reaction, cavities, and a fuzzy appearance of the cortex, but the sensitivity and specificity are low [18] and radiologically detectable changes appear much later than the onset of the infection. MRI is useful as it differentiates necrotic from viable tissues and assesses the extent of infection. It is sensitive for detecting osteomyelitis, but its diagnostic accuracy decreases after recent surgery, when metal implants are present and differentiation between sterile inflammation and still-existing infection is difficult [19–21]. The same applies to three-phase bone scintigraphy: although it is useful when negative, it has a very low specificity in the acute/subacute setting as any recent alteration to the bone will result in a positive outcome [22]. White blood cell (WBC) scintigraphy has been extensively studied for peripheral osteomyelitis and is found to be reliable with high overall accuracy rates [23, 24]. All these studies, however, were conducted on heterogeneous patient groups, including joint prosthesis infection and diabetic feet, and none focused specifically on suspected PTO. The diagnostic value of PET/CT with ^18^F-fluorodeoxyglucose (FDG) for osteomyelitis is still under investigation. This technique has the best properties: easy labelling procedure, available in many centres, and short imaging time. Unfortunately, the problem with FDG is that it is aspecific: it accumulates in healing tissues, in inflammation, and in infection, which has led to a huge variation in reported sensitivity and specificity values for osteomyelitis. Furthermore, no interpretation criteria presently exist as to when to declare an FDG-PET positive or negative for infection.

As a baseline for future research and for the development of a national protocol on posttraumatic osteomyelitis, we conducted this inventory study. Our primary aim was to assess current preferred imaging strategies for diagnosing PTO amongst orthopaedic and trauma surgeons, radiologists, and nuclear medicine physicians. Secondary aims were to determine the preferred serum inflammatory marker for diagnosing PTO and the existence of a local hospital protocol to diagnose and manage PTO.

## Materials and methods

### Participants and data collection

This study utilised an online 16-question survey (the diagnostic osteomyelitis survey) designed to assess individual professionals’ current preferred strategy for diagnosing PTO. Eligible study participants were Dutch consultants and registrars in orthopaedic and trauma surgery, musculoskeletal (MSK) radiology, and nuclear medicine. Requests for participation (followed by two reminders in case of no response) were sent via an email that described the outline of the study and its aim, with an invitation to complete a web-based survey. A total of 2343 invitations were sent to members of the four medical professional associations: the Dutch Society for Trauma Surgery (NVT; 581 invitations), the Dutch Orthopaedic Society (NOV; 1331 invitations), the Dutch Society of Nuclear Medicine (NVNG; 161 invitations), and the musculoskeletal (MSK) section of the Dutch Radiology Society (NVvR; 270 invitations). In The Netherlands, there are 133 hospitals, eight of which are University Medical Centres (UMC) and 28 large peripheral teaching hospitals (PTH) [25]. In this study, the remaining 97 smaller hospitals were regarded as peripheral non-teaching hospitals (PNTH), as the gamut of medical specialist training possibilities at such hospitals is limited or absent.

We developed the web-based diagnostic osteomyelitis survey using the secure Share Point Server 2013 of University Medical Center Groningen (UMCG) in The Netherlands; the survey was presented to the respondents using an https connection. All data were de-identified and stored securely on the UMCG server; access was restricted to the research team. The local UMCG medical ethical committee judged the methods employed in this study and waived further need for approval (reference number METc2014.554).

### The diagnostic osteomyelitis survey

The survey consisted of 16 questions and took approximately 10 min to complete. For some questions, more than one answer option could be selected. Demographic data of the responders were collected, including profession and hospital-specific data.

**Fig. 1:**
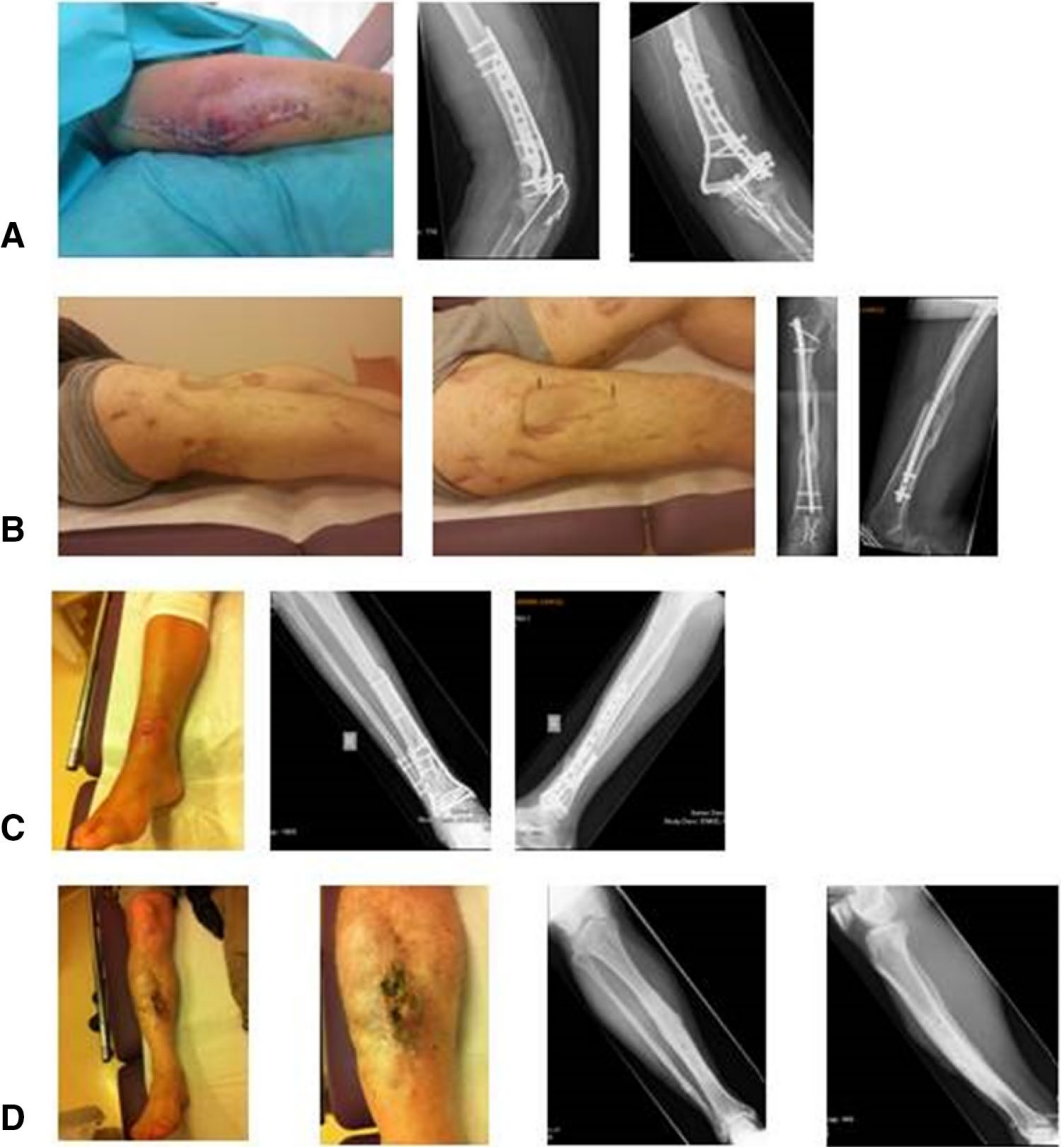
Patient-based clinical scenarios. Patient **A**: A 58-year-old healthy man underwent an open reduction and internal fixation (ORIF) of a comminuted intra-articular distal humerus fracture 1 week ago. The postoperative X-Ray showed an adequate fracture reduction and good position of the osteosynthetic material. After 1 week, a wound infection was diagnosed and it was decided to bring the patient back to theatre for a wound washout. His CRP is 68 mg/l, white cell count 11.5 × 10^9^/l. Patient **B**: A 23-year-old healthy man underwent intramedullary nailing for a Gustillo grade 3B open comminuted femur fracture 1 year ago. The initial stabilization was followed by multiple wound debridements, changing of Vacuum Assisted Closure (VAC) dressings and finally split skin grafting of the wound. During the repeated VAC changes, the nail was palpable in the wound. The patient’s main complaint is pain around the fracture site over the last few months. On examination, there is no wound breakdown. His CRP is 27 mg/l, white cell count 6.5 × 10^9^/l, and ESR 48 mm/hr. Patient **C**: A 44-year-old healthy woman underwent ORIF of a closed comminuted distal tibia fracture 5 months ago. She is referred because the operation wound broke down 2 weeks postoperatively and has not healed since. Her CRP is 3.3 mg/l, white cell count 8.1 × 10^9^/l. Patient **D**: A 49-year-old healthy man underwent multiple operations because of an open fracture of his right tibia and fibula 30 years ago. Although the treatment was complicated by a deep surgical site infection, bone healing was eventually achieved and all metal implants were removed a few years after his last operation. The wound settled down until 18 months ago when an unstable crust developed in the scar. His CRP is 5.9 mg/l, white cell count 8.9 × 10^9^/l. Question after each case description: a) would you order a conventional X-Ray? (Note: X-rays are only provided if the responder selected ‘yes’). b) Would you request further imaging? If yes: please select preferred imaging modality (more than one answer possible, please see “materials and methods” for details)

To assess the current preferred imaging strategies of the responders as realistically as possible, four patient-based clinical cases were presented. Each case described a patient with a different stage of fracture-related osteomyelitis, representing a typical clinical scenario (Fig. 1). These patients gave written consent for the anonymous use of their medical imaging. Each case was introduced with the relevant medical history of the patient combined with a clinical picture of the affected limb. Patient A had an acute surgical site infection (SSI) 1 week after open reduction and internal fixation (ORIF) of a distal humerus fracture. Patients B, C, and D had a suspected (patient B) or obvious (patients C and D) late infection after surgical fracture care. The distinction between patients C and D was the presence of metal implants.

X-rays of the fracture site were only provided if the participants selected the answer option that they would order one. Subsequently, the participants were asked to select which imaging modality they considered most suitable to diagnose or exclude the presence of posttraumatic osteomyelitis (more than one answer was allowed). The imaging options given were: ultrasound, ultrasound-guided biopsy, CT scan with or without intravenous contrast, CT-scan-guided biopsy, MRI scan with or without intravenous contrast, three-phase bone scan with or without SPECT/CT, white blood cell scintigraphy with or without SPECT/CT, and FDG-PET with or without CT. There was also the possibility to provide a personal, non-listed answer.

Participants were also asked which serum inflammatory marker they thought was specific enough to be used for diagnosing PTO (CRP, LC, or ESR) and whether they were aware of a local hospital protocol for diagnosis and management of PTO.

### Data analysis

Data were analysed using IBM SPSS Statistics for Windows (version 22.0, Armonk, NY: IBM Corp.).

## Results

The overall response rate was 15% (*n* = 346); 27% of the trauma surgeons (*n* = 155), 8% of the orthopaedic surgeons (*n* = 102), 35% of the nuclear medicine physicians (*n* = 57) and 12% of the MSK radiologists (*n* = 33) responded. Table 1 presents the responders’ characteristics.

**Table 1:** Responders’ characteristics

	Trauma surgeon (*N* = 153)	Orthopedic surgeon (*N* = 104)	Nuclear physician (*N* = 56)	MSK radiologist (*N* = 33)
Age (years)				
<35	30 (20)	23 (22)	7 (12)	8 (24)
35–50	89 (58)	53 (51)	34 (61)	13 (39)
>50	34 (22)	28 (27)	15 (27)	12 (36)
Medical experience				
Registrar	32 (21)	21 (20)	0 (0)	4 (12)
Consultant	121 (79)	83 (80)	56 (100)	29 (88)
Hospital type				
Non-teaching hospital (*n* = 97)	22 (14)	40 (38)	31 (55)	10 (30)
Peripheral teaching hospital (*n* = 28)	79 (52)	34 (33)	11 (20)	11 (33)
University teaching hospital (*n* = 8)	52 (34)	30 (29)	14 (25)	12 (36)

Data are presented as *N* (%)

The results for the preferred medical imaging modalities for patients A–D are listed in Table 2. There was consensus on the usefulness of a conventional X-Ray in patients with a late infection (patients B, C, and D). In patient A with an early SSI, a repeat X-Ray was requested only 203 times (54.4%); in patients B, C, and D, a conventional X-Ray was requested, respectively, 363 (97.3%), 357 (95.7%), and 351 (94.1%) times.

**Table 2:** Responders’ preferred imaging modalities per patient scenario

	X-Ray	Ultrasound	Ultrasound-guided biopsy	CT-scan without IV contrast	CT-scan with IV contrast	CT-guided biopsy	MRI scan without IV contrast	MRI scan with IV contrast	3-phase bone scan with/without SPECT/CT	WBC scintigraphy with/without SPECT/CT	FDG-PET with/without CT
Patient A											
Trauma surgeon (*N* = 153)	88 (58)	4 (3)	8 (5)	0 (0)	1 (1)	1 (1)	0 (0)	1 (1)	0 (0)	0 (0)	1 (1)
Orthopaedic surgeon (*N* = 104)	65 (63)	11 (11)	14 (14)	2 (2)	1 (1)	0 (0)	0 (0)	2 (2)	1 (1)	2 (2)	0 (0)
Nuclear physician (*N* = 56)	20 (36)	16 (29)	6 (11)	0 (0)	0 (0)	0 (0)	0 (0)	0 (0)	1 (2)	0 (0)	10 (18)
MSK radiologist (*N* = 33)	21 (64)	13 (39)	12 (36)	1 (3)	4 (12)	0 (0)	0 (0)	14 (42)	4 (12)	6 (18)	3 (9)
Patient B											
Trauma surgeon (*N* = 153)	153 (100)	1 (1)	3 (2)	2 (1)	6 (4)	2 (1)	0 (0)	2 (1)	5 (3)	3 (2)	49 (32)
Orthopaedic surgeon (*N* = 104)	102 (98)	3 (3)	5 (5)	36 (35)	9 (9)	8 (8)	2 (2)	6 (6)	23 (22)	33 (32)	8 (8)
Nuclear physician (*N* = 56)	48 (86)	2 (4)	2 (4)	0 (0)	0 (0)	0 (0)	0 (0)	0 (0)	5 (9)	2 (4)	14 (25)
MSK radiologist (*N* = 33)	33 (100)	1 (3)	1 (3)	5 (15)	4 (12)	0 (0)	1 (3)	17 (52)	10 (30)	7 (21)	7 (21)
Patient C											
Trauma surgeon (*N* = 153)	151 (99)	0 (0)	0 (0)	6 (4)	5 (3)	0 (0)	0 (0)	2 (2)	4 (3)	3 (2)	41 (27)
Orthopaedic surgeon (*N* = 104)	103 (99)	3 (3)	2 (2)	37 (36)	9 (9)	6 (6)	5 (5)	6 (6)	26 (25)	23 (22)	8 (8)
Nuclear physician (*N* = 56)	46 (82)	1 (2)	1 (2)	0 (0)	0 (0)	0 (0)	0 (0)	0 (0)	5 (9)	2 (4)	15 (27)
MSK radiologist (*N* = 33)	32 (97)	3 (9)	2 (6)	4 (12)	4 (12)	0 (0)	0 (0)	17 (52)	10 (30)	8 (25)	6 (18)
Patient D											
Trauma surgeon (*N* = 153)	146 (95)	4 (3)	1 (1)	2 (1)	3 (2)	1 (1)	0 (0)	5 (3)	4 (3)	3 (2)	46 (30)
Orthopaedic surgeon (*N* = 104)	104 (100)	2 (2)	1 (1)	12 (12)	10 (10)	6 (6)	15 (14)	41 (39)	26 (25)	23 (22)	8 (8)
Nuclear physician (*N* = 56)	44 (79)	2 (4)	0 (0)	0 (0)	0 (0)	0 (0)	0 (0)	1 (2)	5 (9)	0 (0)	20 (36)
MSK radiologist (*N* = 33)	31 (94)	1 (3)	1 (3)	3 (9)	0 (0)	0(0)	1 (3)	27 (82)	11 (33)	1 (3)	1 (3)

Data are presented as *N* (%)

In those patients with late PTO (patient B, C, and D), there was a remarkable difference between trauma surgeons and orthopaedic surgeons in terms of choice of nuclear medicine imaging. Trauma surgeons favoured FDG-PET, while orthopaedic surgeons preferred the WBC scintigraphy. A similar, consistent difference was seen between radiologists and nuclear medicine physicians regarding the choice for radiology imaging versus nuclear medicine imaging. For example, in patient D, an MRI was favoured by 82% of the MSK radiologists versus 2% of the nuclear medicine physicians. For an FDG-PET/CT for the same patient, these percentages were 3 versus 36%, respectively. CT scans and three-phase bone scans for late fracture-related infections were popular among orthopaedic surgeons and to lesser extent MSK radiologists, though not by trauma surgeons or nuclear medicine physicians. Ultrasound-guided biopsy was regarded by all physicians to have some role in patients with an early infection (patient A), but was not popular for patients with late infections.

The choice for an imaging modality was also influenced by the availability of this technique in the responder’s own hospital. For example, of all those responders who could perform an FDG-PET/CT in their own institution, 21.2% elected it as their preferred imaging method of choice, whereas it was chosen by only 7.2% of responders who did not have an FDG-PET/CT in their own hospital available (Table 3).

**Table 3:** Responder’s preferred imaging modalities per patient scenario corrected for the in hospital available imaging techniques

	X-ray	Ultrasound	Ultrasound-guided biopsy	CT-scan without IV contrast	CT-scan with IV contrast	CT-guided biopsy	MRI scan without IV contrast	MRI scan with IV contrast	3-phase bone scan with/without SPECT/CT	WBC scintigraphy with/without SPECT/CT	FDG-PET with/without CT
Patient A											
Trauma surgeon	88 (56) (*N* = 152)	4 (3) (*N* = 151)	8 (5) (*N* = 151)	0 (0) (*N* = 150)	1 (1) (*N* = 150)	1 (1) (*N* = 150)	0 (0) (*N* = 148)	1 (1) (*N* = 148)	0 (0) (*N* = 19)	0 (0) (*N* = 15)	1 (1) (*N* = 103)
Orthopaedic surgeon	65 (63) (*N* = 103)	10 (10) (*N* = 98)	14 (14) (*N* = 98)	2 (2) (*N* = 96)	1 (1) (*N* = 96)	0 (0) (*N* = 96)	0 (0) (*N* = 96)	2 (2) (*N* = 96)	1 (1) (*N* = 83)	2 (3) (*N* = 72)	0 (0) (*N* = 54)
Nuclear physician	18 (33) (*N* = 54)	16 (30) (*N* = 54)	6 (11) (*N* = 54)	0 (0) (*N* = 55)	0 (0) (*N* = 55)	0 (0) (*N* = 55)	0 (0) (*N* = 52)	0 (0) (*N* = 52)	1 (17) (*N* = 6)	0 (0) (*N* = 6)	10 (21) (*N* = 48)
MSK radiologist	21 (64) (*N* = 33)	13 (39) (*N* = 33)	12 (36) (*N* = 33)	1 (3) (*N* = 33)	4 (12) (*N* = 33)	0 (0) (*N* = 33)	0 (0) (*N* = 33)	14 (42) (*N* = 33)	4 (13) (*N* = 30)	6 (32) (*N* = 19)	3 (12) (*N* = 25)
Patient B											
Trauma surgeon	152 (100) (*N* = 152)	1 (1) (*N* = 151)	3 (2) (*N* = 151)	2 (1) (*N* = 150)	6 (4) (*N* = 150)	2 (1) (*N* = 150)	0 (0) (*N* = 148)	2 (1) (*N* = 148)	5 (26) (*N* = 19)	2 (13) (*N* = 15)	38 (37) (*N* = 103)
Orthopaedic surgeon	101 (98) (*N* = 103)	2 (2) (*N* = 98)	4 (4) (*N* = 98)	34 (35) (*N* = 96)	8 (8) (*N* = 96)	8 (8) (*N* = 96)	2 (2) (*N* = 96)	6 (6) (*N* = 96)	20 (24) (*N* = 83)	28 (39) (*N* = 72)	7 (13) (*N* = 54)
Nuclear physician	47 (87) (*N* = 54)	2 (4) (*N* = 54)	2 (4) (*N* = 54)	0 (0) (*N* = 55)	0 (0) (*N* = 55)	0 (0) (*N* = 55)	0 (0) (*N* = 52)	0 (0) (*N* = 52)	4 (67) (*N* = 6)	2 (33) (*N* = 6)	13 (27) (*N* = 48)
MSK radiologist	33 (100) (*N* = 33)	1 (3) (*N* = 33)	1 (3) (*N* = 33)	5 (15) (*N* = 33)	4 (12) (*N* = 33)	0 (0) (*N* = 33)	1 (3) (*N* = 33)	17 (52) (*N* = 33)	10 (33) (*N* = 30)	6 (32) (*N* = 19)	7 (28) (*N* = 25)
Patient C											
Trauma surgeon	150 (99) (*N* = 152)	0 (0) (*N* = 151)	0 (0) (*N* = 151)	6 (4) (*N* = 150)	5 (3) (*N* = 150)	0 (0) (*N* = 150)	0 (0) (*N* = 148)	2 (2) (*N* = 148)	4 (21) (*N* = 19)	1 (7) (*N* = 15)	33 (32) (*N* = 103)
Orthopaedic surgeon	102 (99) (*N* = 103)	2 (2) (*N* = 98)	1 (1) (*N* = 98)	36 (38) (*N* = 96)	8 (8) (*N* = 96)	6 (6) (*N* = 96)	5 (5) (*N* = 96)	6 (6) (*N* = 96)	23 (28) (*N* = 83)	19 (26) (*N* = 72)	7 (13) (*N* = 54)
Nuclear physician	44 (82) (*N* = 54)	1(2) (*N* = 54)	1 (2) (*N* = 54)	0 (0) (*N* = 55)	0 (0) (*N* = 55)	0 (0) (*N* = 55)	0 (0) (*N* = 52)	0 (0) (*N* = 52)	5 (83) (*N* = 6)	2 (33) (*N* = 6)	14 (29) (*N* = 48)
MSK radiologist	32 (97) (*N* = 33)	3 (9) (*N* = 33)	2 (6) (*N* = 33)	4 (12) (*N* = 33)	4 (12) (*N* = 33)	0 (0) (*N* = 33)	0 (0) (*N* = 33)	17 (52) (*N* = 33)	11 (37) (*N* = 30)	7 (37) (*N* = 19)	6 (24) (*N* = 25)
Patient D											
Trauma surgeon	145 (95) (*N* = 152)	4 (3) (*N* = 151)	1 (1) (*N* = 151)	2 (1) (*N* = 150)	3 (2) (*N* = 150)	1 (1) (*N* = 150)	0 (0) (*N* = 148)	5 (3) (*N* = 148)	5 (26) (*N* = 19)	1 (7) (*N* = 15)	37 (36) (*N* = 103)
Orthopaedic surgeon	103 (100) (*N* = 103)	1 (1) (*N* = 98)	1 (1) (*N* = 98)	12 (13) (*N* = 96)	9 (9) (*N* = 96)	6 (6) (*N* = 96)	15 (16) (*N* = 96)	40 (42) (*N* = 96)	29 (35) (*N* = 83)	20 (28) (*N* = 72)	7 (13) (*N* = 54)
Nuclear physician	42 (78) (*N* = 54)	2 (4) (*N* = 54)	0 (0) (*N* = 54)	0 (0) (*N* = 55)	0 (0) (*N* = 55)	0 (0) (*N* = 55)	0 (0) (*N* = 52)	1 (2) (*N* = 52)	4 (67) (*N* = 6)	0 (0) (*N* = 6)	17 (35) (*N* = 48)
MSK radiologist	31 (94) (*N* = 33)	1 (3) (*N* = 33)	1 (3) (*N* = 33)	3 (9) (*N* = 33)	0 (0) (*N* = 33)	0 (0) (*N* = 33)	1 (3) (*N* = 33)	27 (82) (*N* = 33)	4 (13) (*N* = 30)	0 (0) (*N* = 19)	1 (4) (*N* = 25)

Data are presented as number of responders *n* (%) who would select this imaging modality compared to the total number of responders (*N*) with this imaging modality available in their hospital

None of the serum inflammatory markers was regarded as very specific for diagnosing PTO, but CRP was thought to be the most useful laboratory test and the most popular amongst orthopaedic surgeons (Table 4). One-third of all responders (36%, *n* = 124) reported being aware of a hospital protocol for the treatment of osteomyelitis; the other responders were either unaware of a protocol (25%, *n* = 86) or reported an absence of one (39%, *n* = 136) (Table 5). The availability of a PTO protocol was the highest in the University Medical Centres (Table 6).

**Table 4:** Preferred serum inflammatory markers for diagnosing PTO

	C-reactive protein	Leukocyte count	Erythrocyte sedimentation rate
Trauma surgeon (*N* = 153)	86 (56)	47 (31)	63 (41)
Orthopaedic surgeon (*N* = 104)	74 (71)	26 (25)	55 (53)
Nuclear medicine physician (*N* = 56)	29 (52)	20 (36)	16 (29)
MSK radiologist (*N* = 33)	14 (42)	14 (42)	12 (36)

Result of the question: ‘Which serum inflammatory marker do you regard useful for diagnosing PTO’? Note: more than one answer was possible

**Table 5:** Availability of PTO protocol per medical specialty

Medical specialty	Frequency	Percent
Trauma surgeon (*N* = 153)		
Yes	51	33
No	71	46
Unsure	31	20
Orthopedic surgeon (*N* = 104)		
Yes	47	45
No	41	39
Unsure	16	15
Nuclear medicine physician (*N* = 56)		
Yes	13	23
No	15	27
Unsure	28	50
MSK radiologist (*N* = 33)		
Yes	13	39
No	9	27
Unsure	11	33

**Table 6:** Availability of PTO protocol per hospital type

Type of hospital	Frequency	Percent
Non-teaching hospital (*N* = 103)		
Yes	23	22
No	50	49
Unsure	30	29
Teaching hospital (*N* = 35)		
Yes	45	33
No	59	44
Unsure	31	23
University medical centre (*N* = 8)		
Yes	56	52
No	27	25
Unsure	25	23

## Discussion

This study confirms the variety in diagnostic strategies that many clinicians dealing with PTO will recognise from their day-to-day practice. Although the overall response rate of our survey was only 15%, the responders are a typical reflection of those working with this patient group (Table 1). One should also keep in mind that it is only a small percentage of all trauma and orthopaedic surgeons who are involved in osteomyelitis care and that we addressed the whole group. Because it is likely that the responders will have an interest in—and, therefore, deeper knowledge of—PTO compared to non-responders, this study is prone to even underestimate the real variety in diagnostic imaging strategies as the first diagnostic manoeuvres might be initiated by the primary surgeon. We, therefore, regard the contribution from 346 medical practitioners as a substantial response and the outcome of this survey as a relevant finding to report to our peers.

The variation in diagnostic workup of patients with suspected PTO is in concordance with the lacking guidelines on this subject and also with the apparent struggle of various authors to formulate clear and practical recommendations. Termaat et al. published a meta-analysis on optimal imaging modalities for chronic osteomyelitis [18]. They concluded that FDG-PET was the most accurate imaging option to diagnose chronic osteomyelitis, with a sensitivity and specificity of 96 and 91%, respectively. However, the paper was published in 2005 and includes studies published between 1975 and 2003. Considering that current medical technology is developing at an almost exponential rate, it is safe to assume that the diagnostic capacities of the different imaging modalities described are no longer truly represented by the papers analysed for that study (e.g., the commercial system to combine PET with CT (PET/CT) first reached the market in 2001 [26]). The data in that paper should, therefore, be interpreted cautiously. Also based on the best available evidence, but still a result from a consensus meeting, is the report of the European Association of Nuclear Medicine (EANM) published in 2014. In this paper, Jutte et al. proposed a diagnostic flowchart for peripheral bone osteomyelitis, including sternal infections [14]. This flowchart is probably the best available tool for clinicians at the moment, but it is a very broad algorithm with an emphasis on nuclear imaging. In the present study, recommendations of this EANM consensus document were not followed by the majority of responders in any of the scenarios presented.

Part of the variance in diagnostic imaging strategies for PTO can be explained from the imaging techniques locally available to the requesting (or advising) medical practitioner (Table 3). Responders tended to favour an imaging modality when this was available in their hospital. Although this is an understandable pragmatic choice, it may not be the most cost-effective strategy. Having an evidence-based guideline for diagnosing (and excluding) PTO will support a radiology and/or nuclear medicine department in negotiating the purchase of future appropriate medical imaging equipment.

Yet another possible explanation for the variance between the subgroups is that, in The Netherlands, the majority of fractures are treated by trauma surgeons who are trained as general surgeons, as opposed to orthopaedic surgeons (66 versus 34%, respectively, as reported in a recent study on hip fractures [27]). Orthopaedic surgeons are more familiar with the (more researched) concept of prosthetic joint infections (PJI), and some of their choices for diagnosing PTO might be extrapolated from these papers. Dutch trauma surgeons, however, focus solely on fractures and are not influenced by previous knowledge on diagnosing PJI, therefore, they might have a different approach to diagnosing fracture-related infections. The same can be said for radiologists versus nuclear medicine physicians—both are highly trained in medical imaging options for various infectious conditions—but they are likely biased by background knowledge of their own area of expertise. This bias does explain the difference in preferred imaging modalities (e.g., MRI versus FDG-PET/CT) in patients with late-onset PTO.

In addition, surgical clinicians and advising imaging specialists often have a different starting point when additional imaging has to be chosen. The clinical situation plays a crucial role in the decision-making process, and nuclear imaging specialists and radiologists have the disadvantage of not being able to examine patients themselves. In this study, the provided clinical patient scenarios were the same for all participating medical specialists, but the difference in background knowledge might have lead to a different imaging strategy. More in general, failure from the surgeon to communicate the essential clinical details and specific diagnostic question with the advising imaging specialist can result in a less logical imaging advice. Another important factor that needs to be emphasised is that the process of treating fracture-related infections is time-consuming and costly. The best available data for this are derived from studies in patients with a prosthetic joint infection (PJI) and diabetic feet. In infected total hip arthroplasties (THAs), for example, the hospital length of stay has been shown to be 2.2 times longer, with associated overall costs 3.1 times higher compared to non-infected primary THA procedures [28, 29]. Non-medical costs resulting from the inability to work and help required from carers are not known. Any delay in diagnosis will obviously also delay the start of treatment and subsequently the recovery of a patient with PTO, hence overall costs will increase. It is known from other orthopaedic studies that, as a general rule, both patients and cost effectiveness benefit from clinical pathways and guidelines [30, 31]. There is thus a need for a lean and strict algorithm on diagnosing PTO. This will help clinicians choose the most effective diagnostic pathway to reduce the time needed to properly diagnose PTO and subsequently reduce medical costs by avoiding unnecessary imaging requests. Our results lead us to believe that in some cases, a leaner diagnostic pathway could have been followed. For example, for the two patients with a late clinical wound breakdown, therefore, a clear infective component (patients C and D), 44% (patient C, *n* = 152) and 43% (patient D, *n* = 149) of all participants would request further imaging, which is mainly indicated to diagnose or exclude an infection (a bone scan, WBC scintigraphy or FDG-PET). Especially for patient D (an obvious infection, no hardware in situ, and all operations performed three decades ago), one could argue that it is more logical to request an imaging modality that will aid in determining the surgical strategy and not only confirm the diagnosis of osteomyelitis. An MRI scan to visualise the extent of the osteomyelitis, and the presence or absence of cloacae, sinuses, subcortical abscesses, and intramedullary sequesters would in this case be a more logical option and is much cheaper and easier to perform than, for example, a WBC scintigraphy or FDG-PET. It is in this perspective interesting to note that an MRI for patient D was selected by only 26% (*n* = 90) of the responders.

The present study was designed to assess current practice on diagnostic imaging strategies for posttraumatic osteomyelitis in The Netherlands. The results will be used as a baseline for the development of a multicentre prospective trial to eventually provide and implement evidence-based national and international guidelines on diagnosing PTO. These guidelines will hopefully decrease the time to diagnosis in a cost-effective way.

### Limitations of this study

This study might be limited due to bias resulting from under-coverage and non-response. Because it is likely that the responders will have an interest in—and, therefore, deeper knowledge of—PTO compared to non-responders, this study is prone to underestime the real variety in diagnostic imaging strategies. A second limitation might be the fact that this study was undertaken in only one European country. However, since no international guidelines on this topic exist, it is likely that the lacking consensus on how to diagnose PTO is an international omission and that our results can be extrapolated to other trauma orthopaedic societies.

## Conclusions

There is no agreement amongst Dutch trauma and orthopaedic surgeons, radiologists, and nuclear physicians regarding the optimal diagnostic strategies to diagnose or exclude posttraumatic osteomyelitis. None of the serum inflammatory markers was regarded as very specific for diagnosing PTO, but CRP was thought to be the most useful laboratory test. The availability and awareness towards local protocols to diagnose and treat PTO are poor. The results of this study support the need for future randomised controlled trials on optimal diagnostic strategies for PTO. There is also a necessity for the development of national and international guidelines on this topic, in which cost effective strategies are based on the best available evidence.
